# Supplementary material for: Transformation of tenofovir into stable ProTide nanocrystals with long-acting pharmacokinetic profiles
Source: Nat Commun. 2021 Sep 16;12:5458. doi: 10.1038/s41467-021-25690-5 (PMC8445934; doi:10.1038/s41467-021-25690-5)
Supplement: Supplementary file 1 — Supplementary Information [file 41467_2021_25690_MOESM1_ESM.pdf]

**Supplementary Information**

# Transformation of tenofovir into stable ProTide nanocrystals with long-acting pharmacokinetic profiles

Denise A. Cobb[1], Nathan Smith[1], Suyash Deodhar [1], Aditya N. Bade[1], Nagsen Gautam[2], Bhagya Laxmi Dyavar Shetty[1], JoEllyn McMillan[1], Yazen Alnouti[2], Samuel M. Cohen[3], Howard E. Gendelman[1,2] Benson Edagwa[1,*]

[1]*Department of Pharmacology and Experimental Neuroscience, University of Nebraska Medical Center, Omaha, NE 68198 USA*

[2]*Department of Pharmaceutical Sciences, University of Nebraska Medical Center, Omaha, NE 68198 USA*

[3]*Department of Pathology and Microbiology, University of Nebraska Medical Center, Omaha, NE 68198 USA*

***Corresponding author:** Benson Edagwa, Ph.D., Department of Pharmacology and Experimental Neuroscience, University of Nebraska Medical Center, Omaha, NE. 68198-5880; phone 402 559 0856; fax 402 559 7495; email benson.edagwa@unmc.edu ORCID: https://orcid.org/0000-0003-1484-9331

## Supplementary Figures

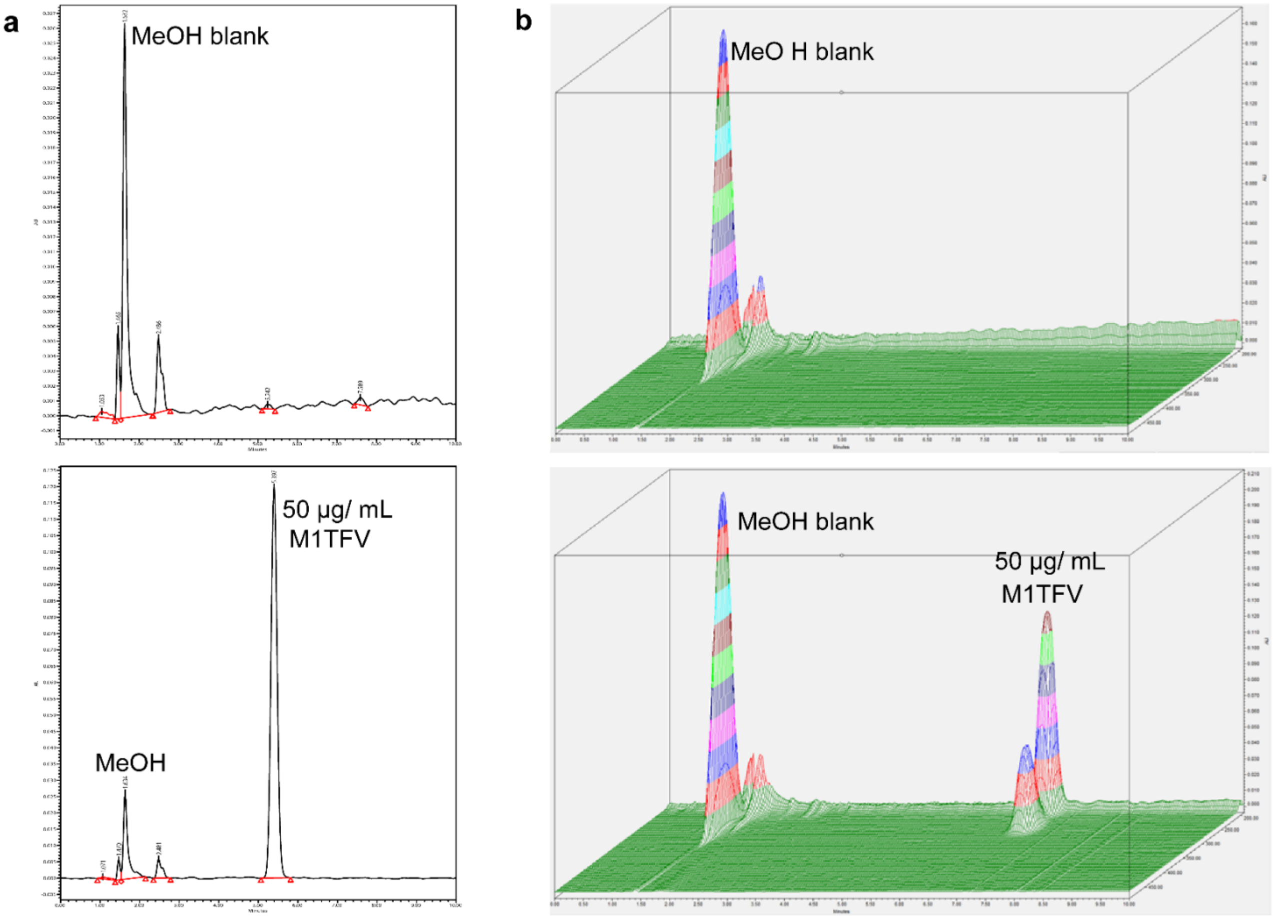


**c**

| Drug Purity Quantitation | | | | | | |
|---|---|---|---|---|---|---|
| Drug | Sr. No. | Total area of peaks | Area of drug peak | % drug peak area | Average % | SEM |
| M1TFV | 1 | 1335436 | 1324828 | 99.21 | 98.37 | 0.63 |
| | 2 | 1361139 | 1322099 | 97.13 | | |
| | 3 | 1364707 | 1347801 | 98.76 | | |

**Supplementary Figure 1. M1TFV Chemical Purity. (a)** A Waters H-Class UPLC coupled to a UV/Vis detector was employed to quantitatively determine the purity of M1TFV. **(b)** A Waters H-Class UPLC coupled to PDA detector provided qualitative assessment of compound purity. **(c)** Purity (%) was calculated by comparing the total peak area (excluding solvent peaks) to the drug peak area observed when **λ=** 210 nm.

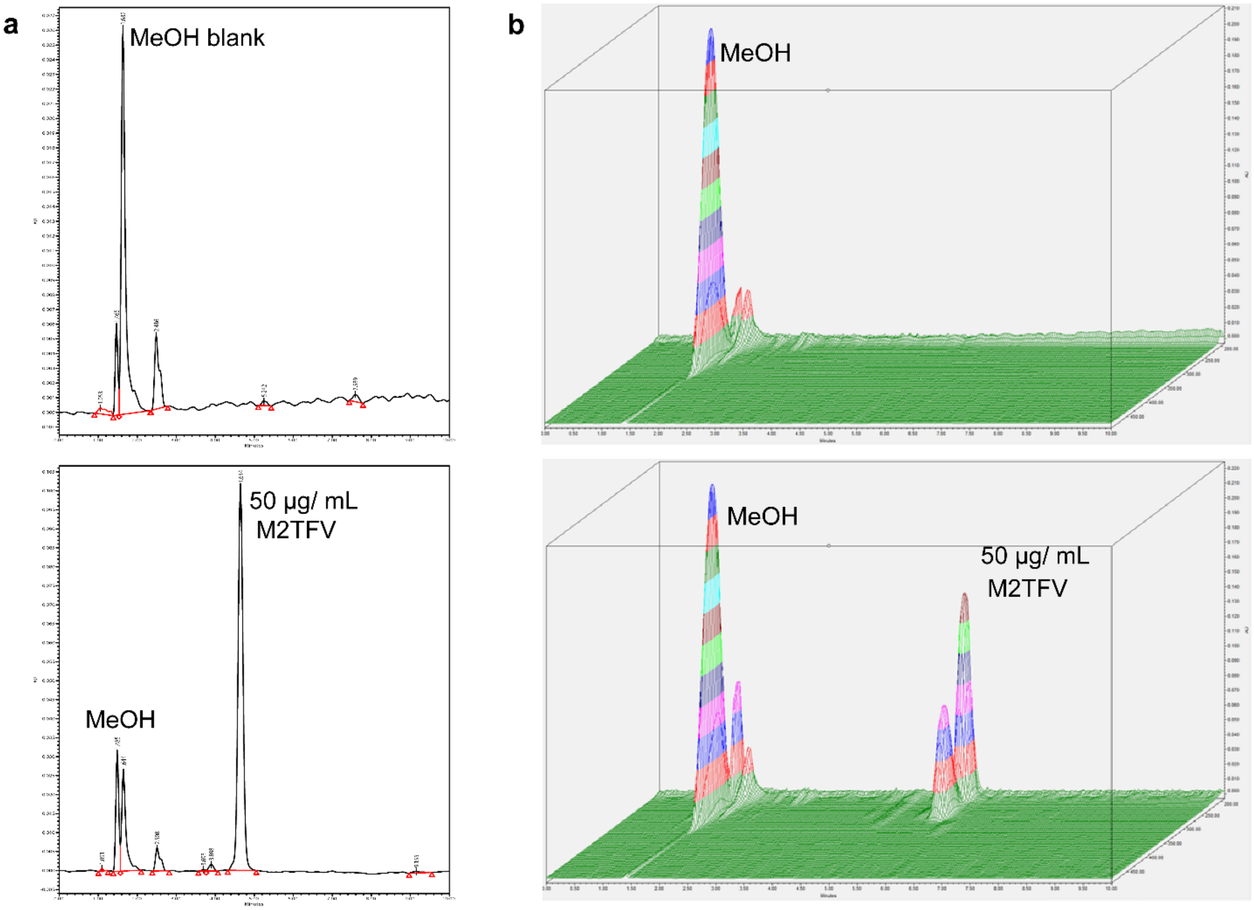


c

| Drug Purity Quantitation | | | | | | |
|---|---|---|---|---|---|---|
| Drug | Sr. No. | Total area of peaks | Area of drug peak | % drug peak area | Average % | SEM |
| M2TFV | 1 | 939642 | 912412 | 97.10 | 94.96 | 1.08 |
| | 2 | 977544 | 919759 | 94.09 | | |
| | 3 | 950840 | 890780 | 93.68 | | |

**Supplementary Figure 2. M2TFV Chemical Purity. (a)** A Waters H-Class UPLC coupled to a UV/Vis detector was employed to quantitatively determine the purity of M2TFV. **(b)** A Waters H-Class UPLC coupled to PDA detector provided qualitative assessment of compound purity. **(c)** Purity (%) was calculated by comparing the total peak area (excluding solvent peaks) to the drug peak area observed when **λ=** 210 nm.

**a** **b**

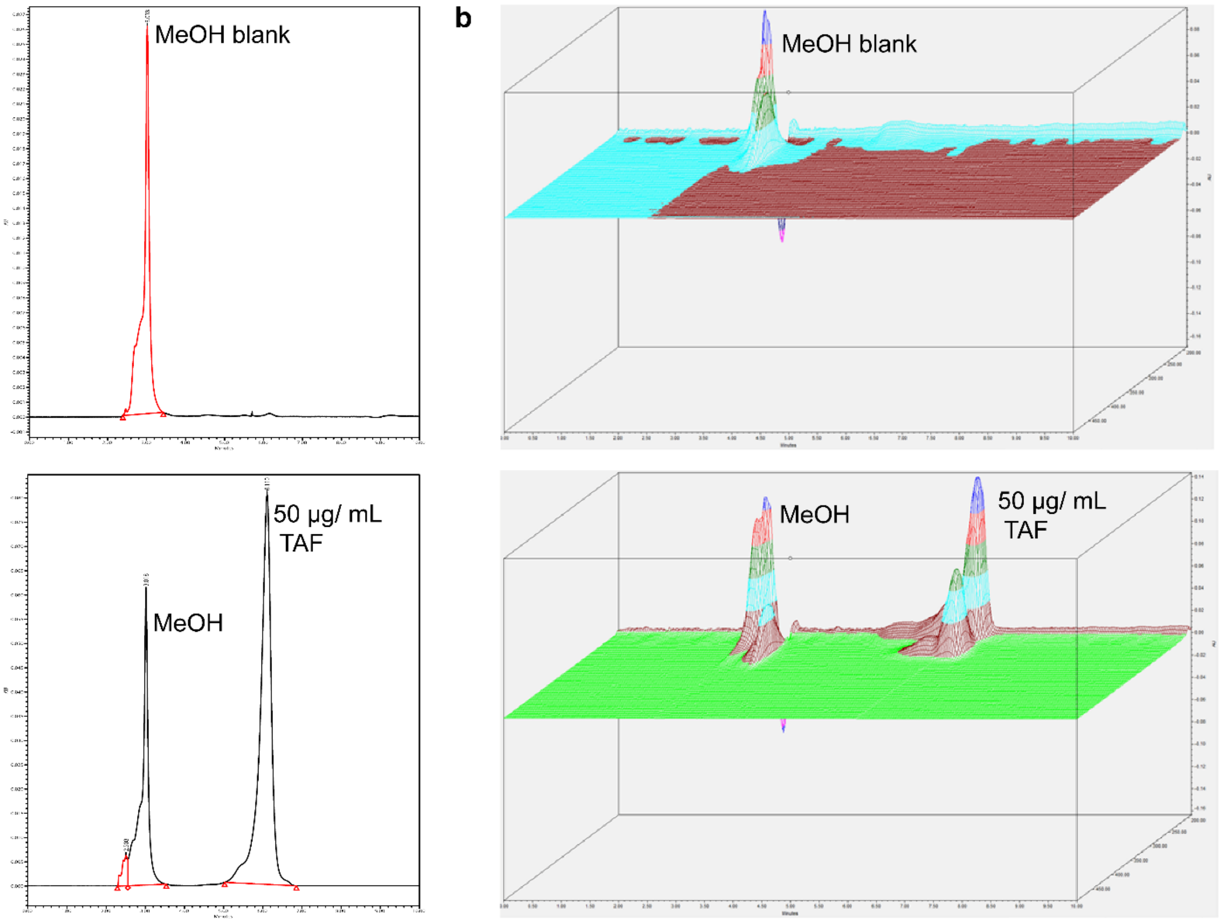


**c**

| Drug Purity Quantitation | | | | | | |
|---|---|---|---|---|---|---|
| Drug | Sr. No. | Total area of peaks | Area of drug peak | % drug peak area | Average % | SEM |
| TAF | 1 | 1590002 | 1590002 | 100.00 | 100.00 | 0.00 |
| | 2 | 1581396 | 1581396 | 100.00 | | |
| | 3 | 1591703 | 1591703 | 100.00 | | |

**Supplementary Figure 3. TAF Chemical Purity. (a)** A Waters H-Class UPLC coupled to a UV/Vis detector was employed to quantitatively determine the purity of TAF. **(b)** A Waters H-Class UPLC coupled to PDA detector provided qualitative assessment of compound purity. **(c)** Purity (%) was calculated by comparing the total peak area (excluding solvent peaks) to the drug peak area observed when **λ=** 260 nm.

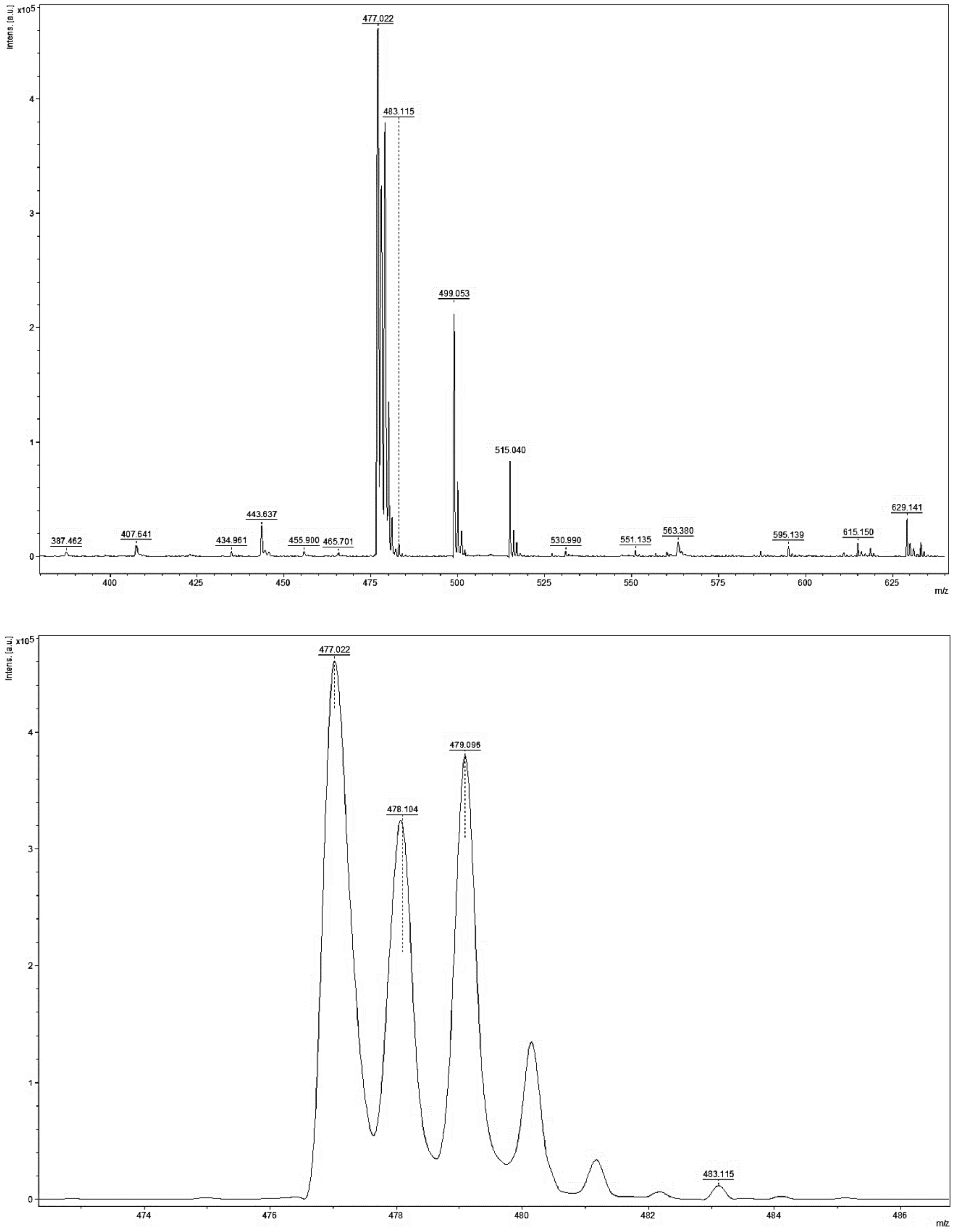


**Supplementary Figure 4. High-Resolution Mass, TAF.** Analysis of TAF by an Autoflex maX MALDI-TOF/TOF mass spectrometer generated a strong signal at 477.022 m/z.

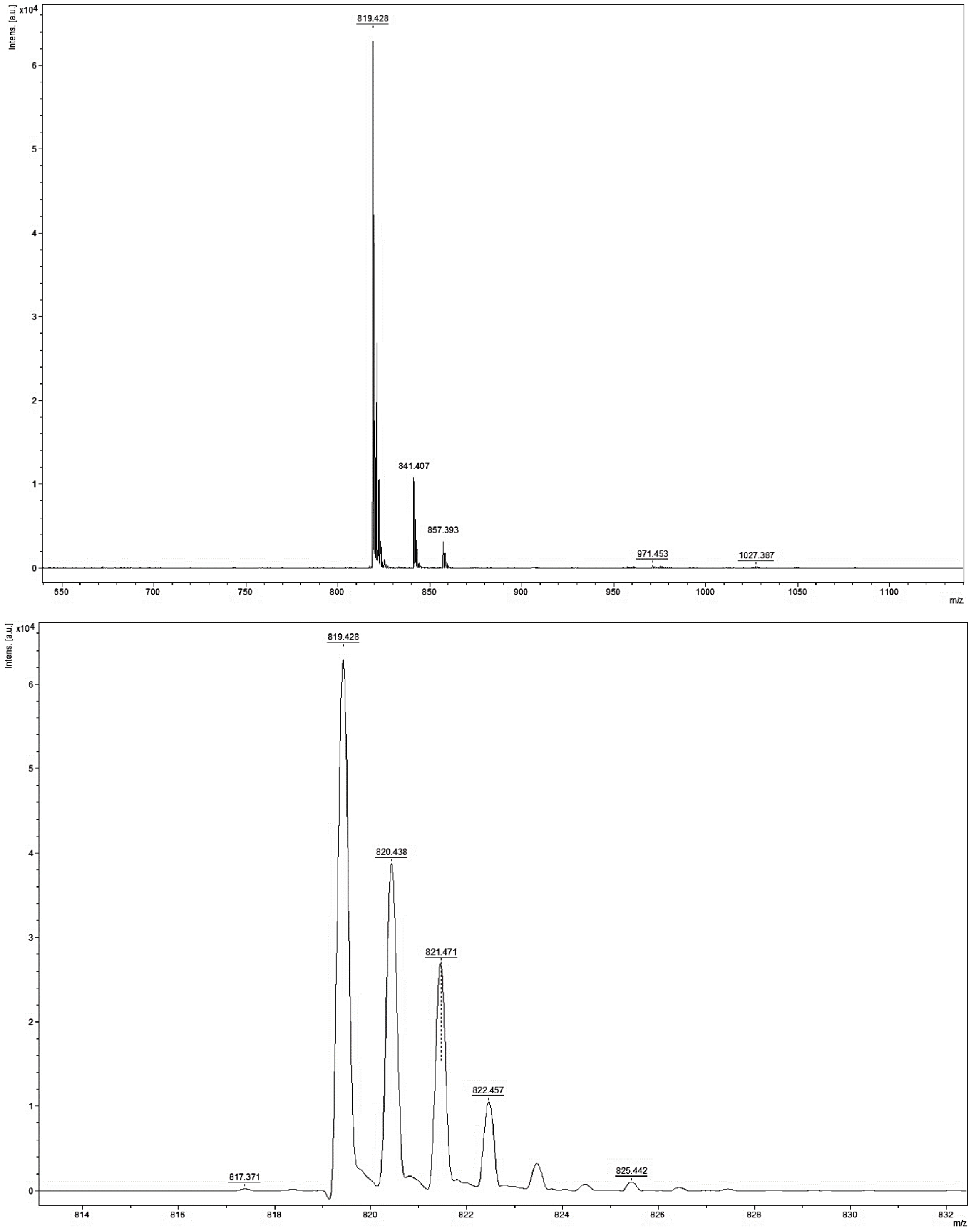


**Supplementary Figure 5. High-Resolution Mass, M1TFV.** Analysis of M1TFV by an Autoflex maX MALDI-TOF/TOF mass spectrometer generated a strong signal at 819.42 m/z.

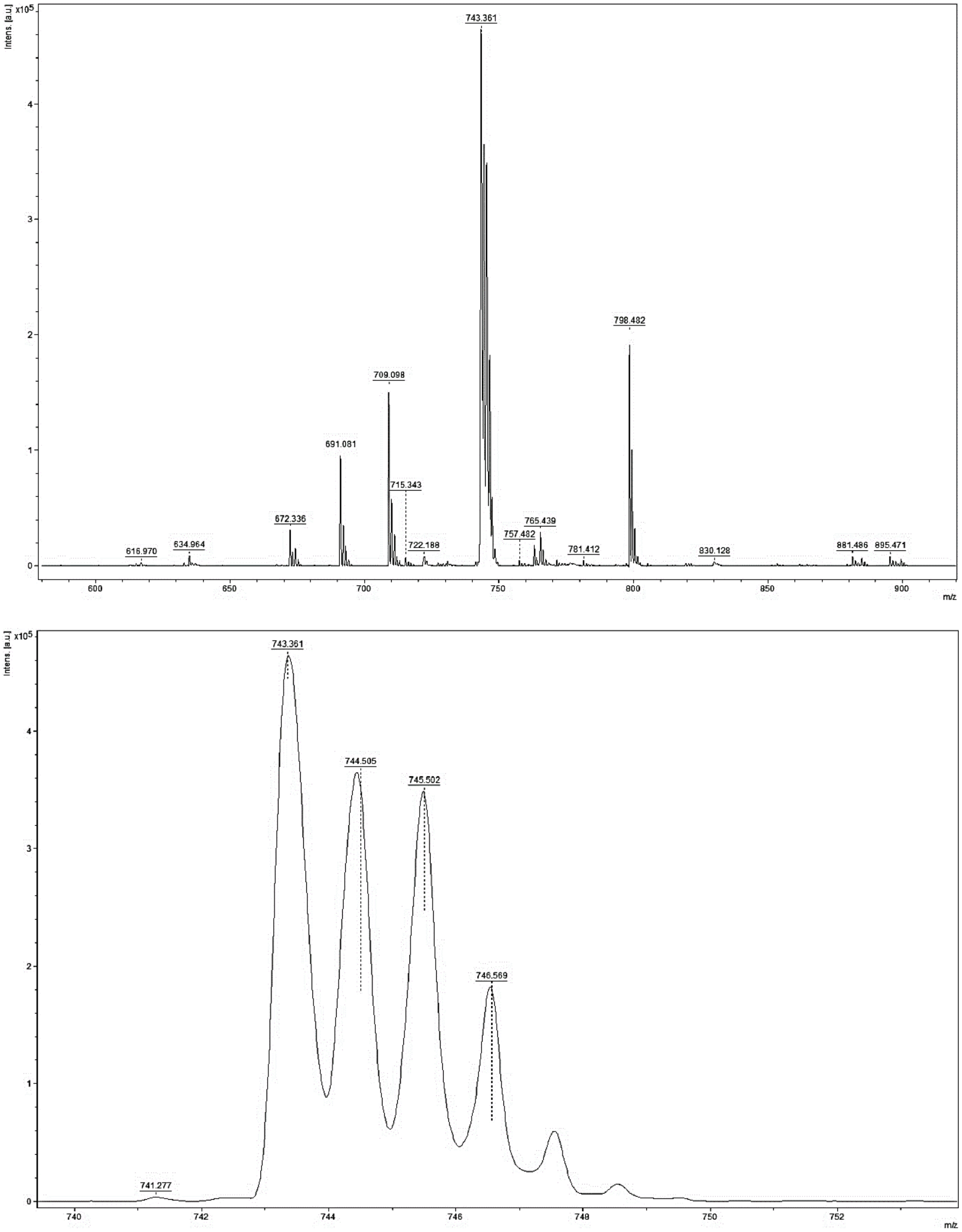


**Supplementary Figure 6. High-Resolution Mass, M2TFV.** Analysis of M2TFV by an Autoflex maX MALDI-TOF/TOF mass spectrometer generated a strong signal at 743.361 m/z.

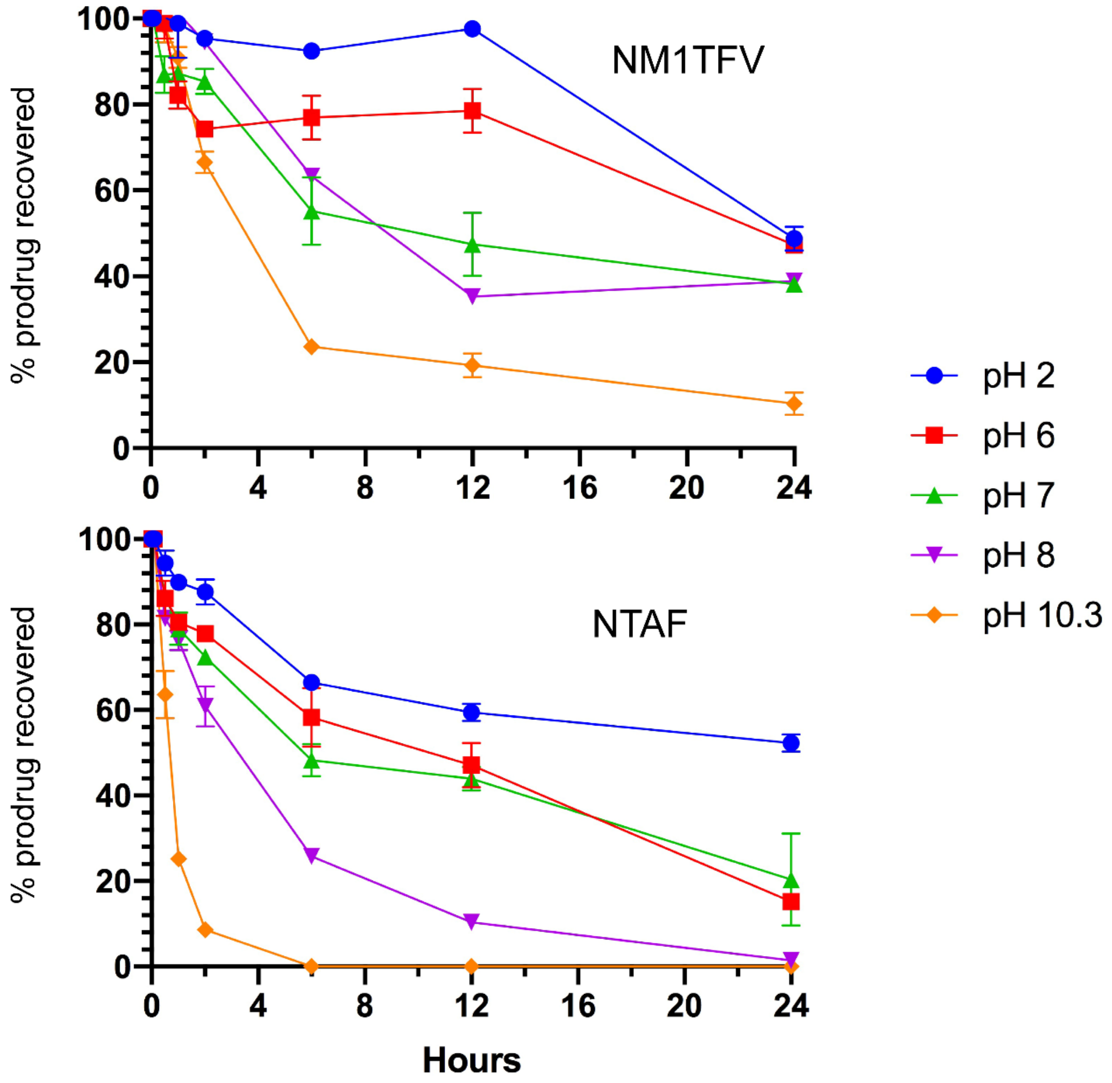


**Supplementary Figure 7. ProTide Physical Stability.** The stability of NM1TFV and NTAF at pH 2, 6, 7,8, and 10.3 was assessed and measured for loss of prodrug over a 24 hour period.

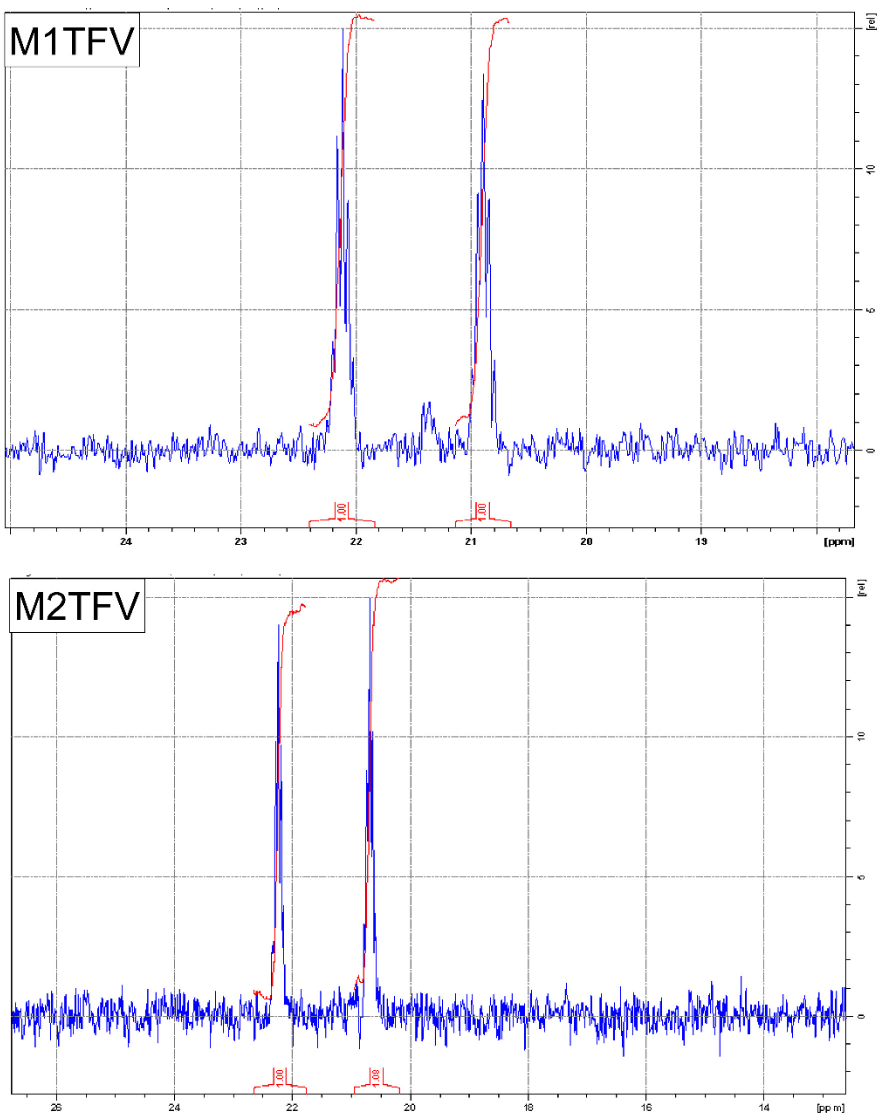


**Supplementary Figure 8. $^{31}$P-NMR.** Phosphorus NMR confirmed the synthesis of M1TFV (above) and M2TFV (below). Peaks in the phosphorous NMR spectra indicated a 1:1 mixture of the R and S stereoisomers for both ProTides.

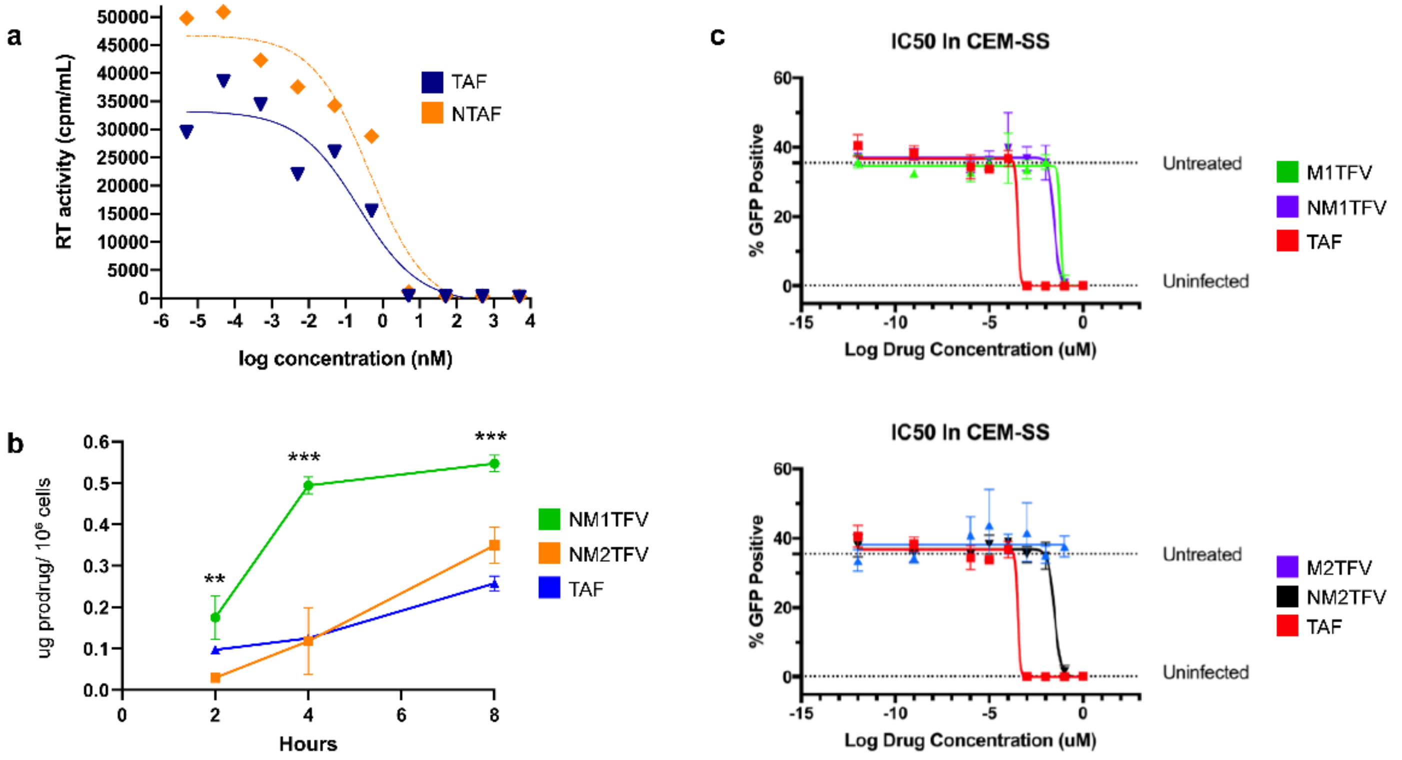


| $IC_{50}$ in MDM | |
|---|---|
| TAF | 0.22 nM |
| NTAF | 0.44 nM |
| **$IC_{50}$ in CEM-SS T-cells** | |
| TAF | 0.332 nM |
| M1TFV | 61.55 nM |
| NM1TFV | 28.81 nM |
| NM2TFV | 30.07 nM |

**Supplementary Figure 9. ProTide Biological Characterizations. (a)** $IC_{50}$ of, TAF and NTAF in MDM and **(b)** Uptake of NM1TFV, NM2TFV, and TAF was determined in CEM-SS T-cells. A one-way analysis of variance (ANOVA) followed by Tukey's post hoc test was used to compare the prodrug and TFV-DP levels among three treatment groups (*$P \leq 0.05$, **$P < 0.01$, ***$P < 0.001$, ****$P < 0.0001$ NM1TFV compared with NM2TFV). **(c)** $IC_{50}$ of M1TFV, M2TFV, NM1TFV, NM2TFV, and TAF were investigated in CEM-SS T-cells at a range of concentrations (0.0001–1000 nM) and determined by HIV-1 reverse transcriptase (RT) activity after viral challenge with HIV-$1_{ADA}$ at an MOI of 0.1. Data was normalized and expressed as percentage of HIV-1 control ± SEM; N = 4.

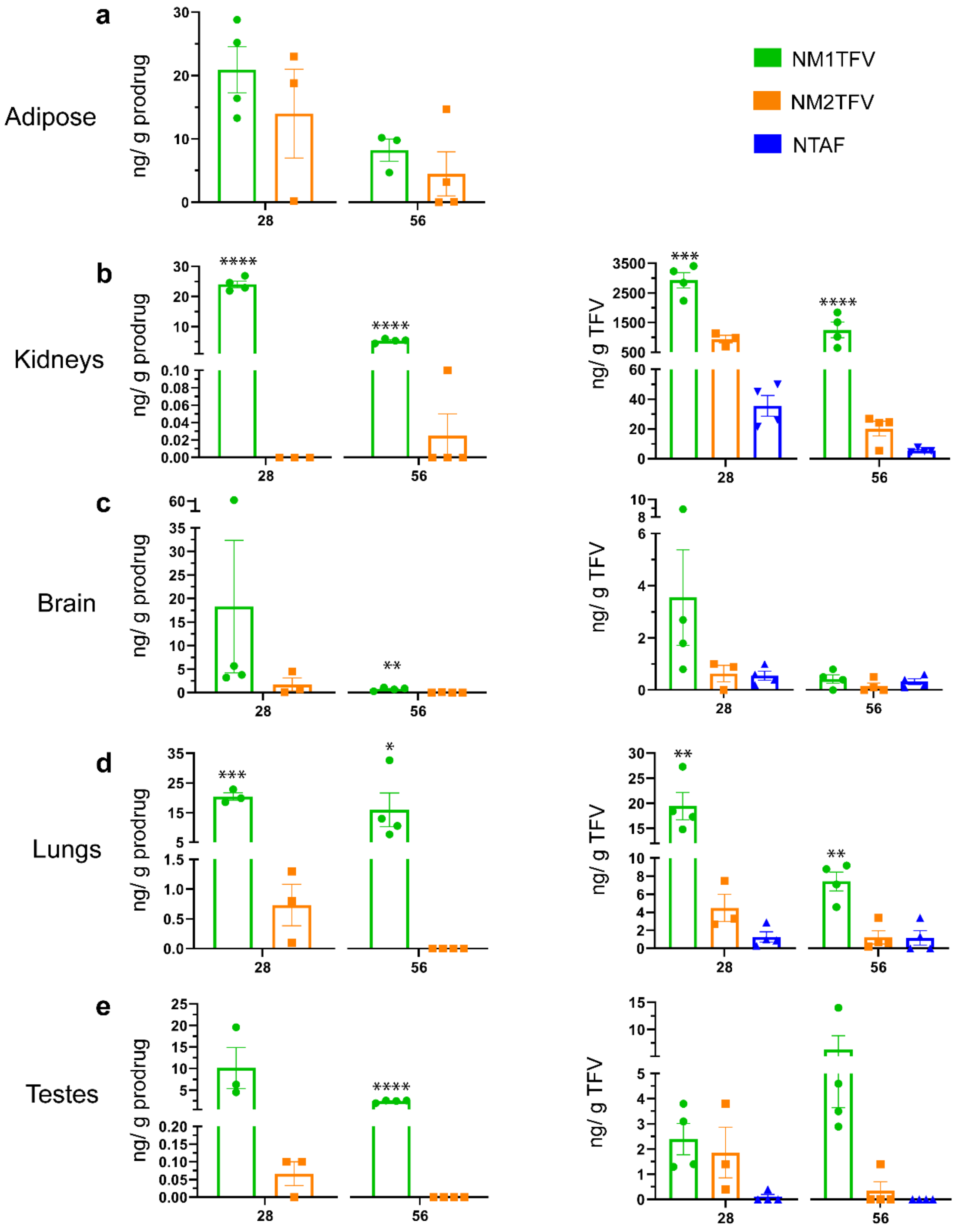


**Supplementary Figure 10. Tissue Biodistribution.** SD rats were administered a single IM dose of NM1TFV, NM2TFV or NTAF (75 mg/kg TFV-eq.) to determine pharmacokinetic (PK) profiles. Tissue biodistribution of M1TFV/ M2TFV and TFV was assessed at 28 and 56 days after injection in the **(a)** adipose **(b)** kidneys (*****P*< 0.0001 NM1TFV vs. NM2TFV prodrug; ****P* = 0.0007 , *****P* <0.0001 NM1TFV vs. NM2TFV TFV), **(c)** brain (***P*=0.0057 NM1TFV vs. NM2TFV prodrug), **(d)** lungs (****P* =0.0001, **P* =0.0303 NM1TFV vs. NM2TFV prodrug; ***P* =0.0017, ***P* = 0.0018 , NM1TFV vs. NM2TFV TFV), and **(e)** testes (*****P* <0.0001 NM1TFV vs. NM2TFV prodrug). Data is expressed as mean ± SEM where *N* = 4 biological replicates. Prodrug concentrations were compared using an unpaired two-tailed *t*-test (*****P* < .0001, ****P* < .001, ***P* < .01, **P* < .05 NM1TFV compared with NM2TFV). To compare TFV levels between three or more groups, ordinary one-way ANOVA followed by Tukey's post hoc test with a single pooled variance (*****P* < .0001, ****P* < .001, ***P* < .01, **P* < .05 NM1TFV compared with NM2TFV).

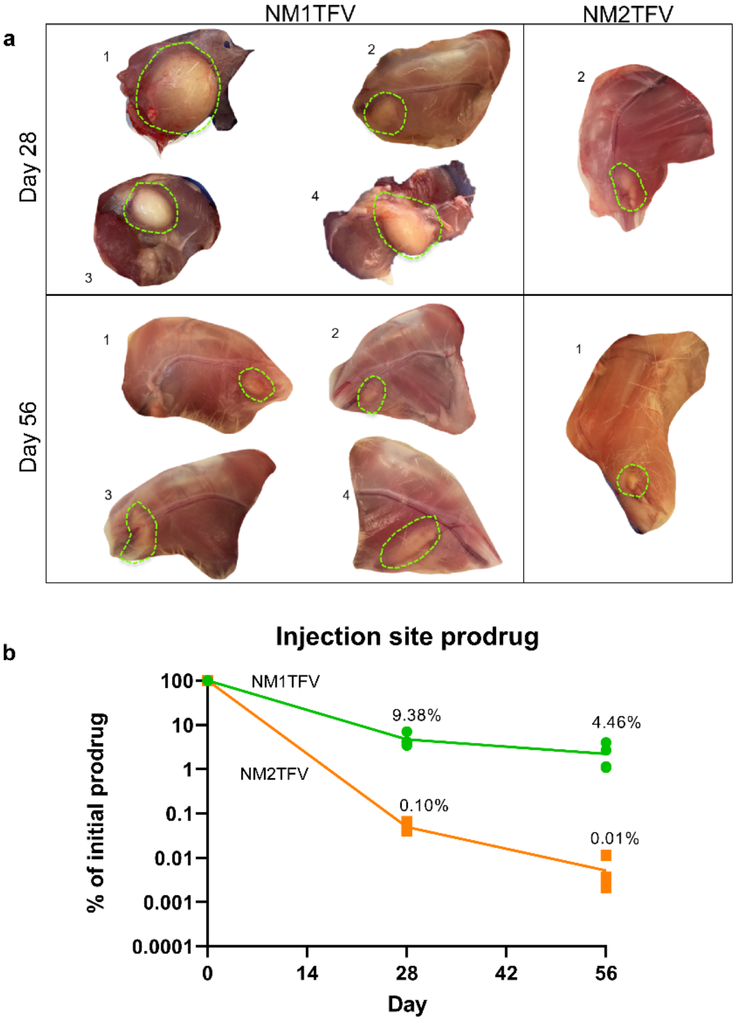


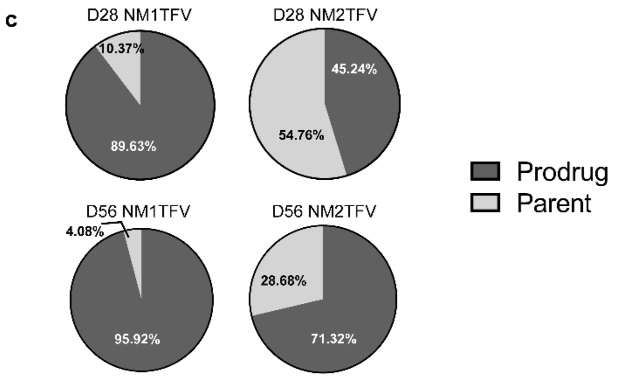


d

| Statistically-significant correlations between muscle prodrug and tissue **prodrug** levels | | | | |
|---|---|---|---|---|
| X | Y | r | $r^2$ | *P* value |
| Liver prodrug | Muscle prodrug | 0.7601 | 0.5778 | 0.0016 |
| Spleen prodrug | Muscle prodrug | 0.8158 | 0.6656 | 0.0002 |
| Lung prodrug | Muscle prodrug | 0.6306 | 0.3977 | 0.0156 |
| Whole blood prodrug | Muscle prodrug | 0.6208 | 0.3854 | 0.0135 |
| Gut prodrug | Muscle prodrug | 0.7520 | 0.5655 | 0.0012 |
| Adipose prodrug | Muscle prodrug | 0.5430 | 0.2948 | 0.0448 |
| Testes prodrug | Muscle prodrug | 0.5977 | 0.3572 | 0.0240 |
| Statistically-significant correlations between muscle prodrug and tissue **TFV** levels | | | | |
| X | Y | r | $r^2$ | *P* value |
| Liver TFV | Muscle prodrug | 0.8503 | 0.7230 | 0.0001 |
| Spleen TFV | Muscle prodrug | 0.9228 | 0.8516 | <0.0001 |
| Lung TFV | Muscle prodrug | 0.8847 | 0.7827 | <0.0001 |
| Whole blood TFV | Muscle prodrug | 0.6981 | 0.4873 | 0.0055 |
| LN TFV | Muscle prodrug | 0.9018 | 0.8132 | <0.0001 |
| Kidney TFV | Muscle prodrug | 0.8109 | 0.6576 | 0.0002 |
| Gut TFV | Muscle prodrug | 0.8242 | 0.6793 | 0.0002 |

**Supplementary Figure 11. Site of injection.** SD rats were administered a single IM dose of NM1TFV, NM2TFV or NTAF (75 mg/kg TFV-eq.) and sacrificed on days 28 and 56 post treatment. (a) Visible intramuscular depots were observed for 4/4 animals treated with NM1TFV, ¼ animals treated with NM2TFV, and 0/4 NTAF animals at day 28. Similarly at day 56, intramuscular depots were observed for 4/4 animals treated with NM1TFV, ¼ animals treated with NM2TFV, and 0/4 NTAF. Green dashed regions indicate the intramuscular drug depot. (b) Concentration of prodrug at the site of injection over the course of the study was measured. Individual muscle prodrug concentrations of 4 animals are plotted, values are expressed as the percent of initial dose is indicated at each time point. (c) Prodrug to parent drug ratios were determined on days 28 and 56. (d) Muscle prodrug concentrations of individual NM1TFV and NM2TFV treated animals (*X*) was plotted against the corresponding tissue drug concentration (*Y*) for individual animals (NM1TFV and NM2TFV), and two-tailed Pearson correlation used to assess the relationship between the two variables, (α= 0.05, ****$P$ < .0001, ***$P$ < .001, **$P$ < .01, *$P$ < .05).

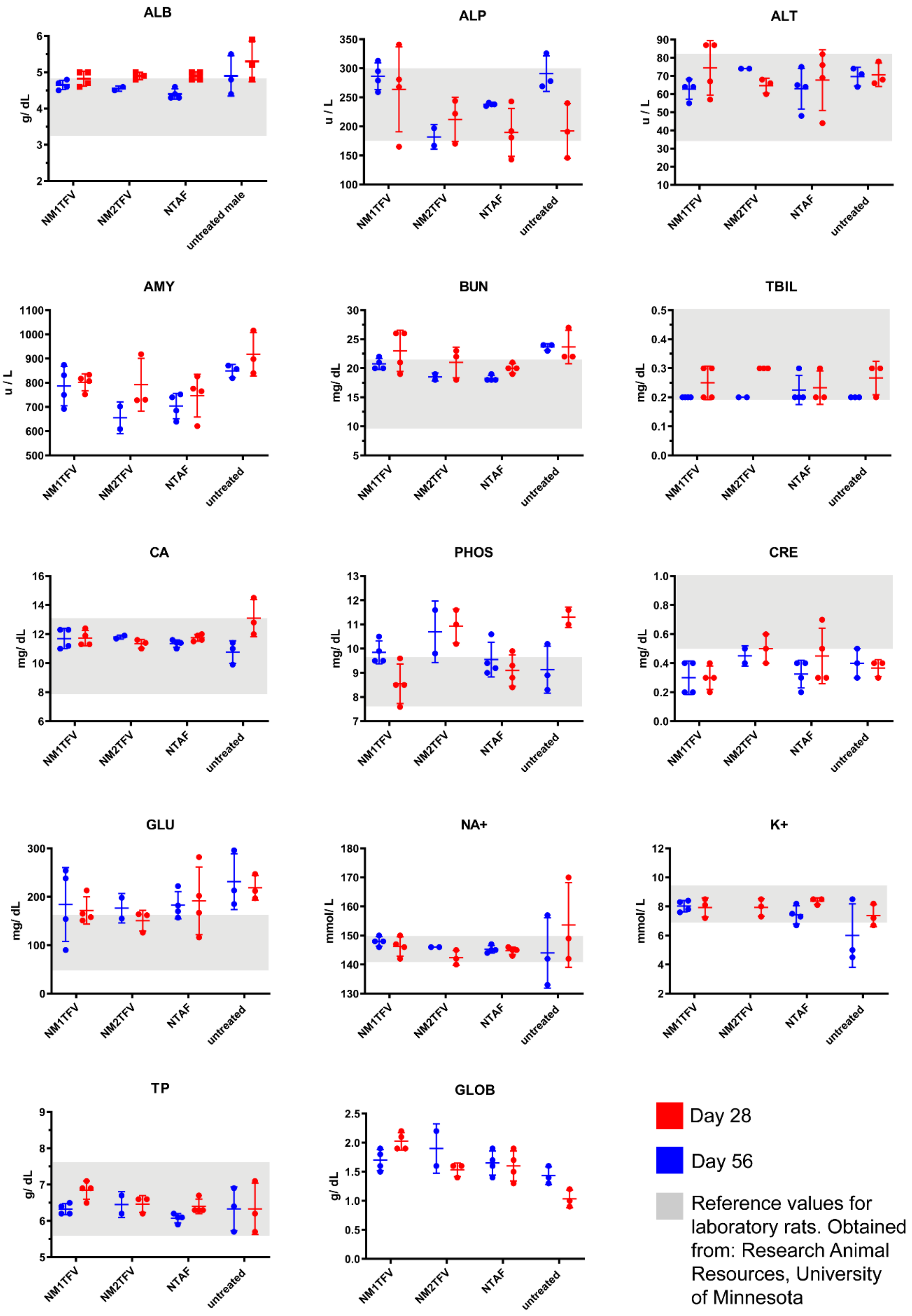


**Supplementary Figure 12. Serum Chemistry.** Toxicity was assessed in SD rats after NM1TFV, NM2TFV, and NTAF treatment. Comprehensive serum chemistry profiles determined on the days of sacrifice (days 28 in red, and 56 in blue), values reported are the SEM of at least 3 replicates. Grey rectangles indicate reference laboratory values for rats, obtained via University of Minnesota, research animal resources.

a

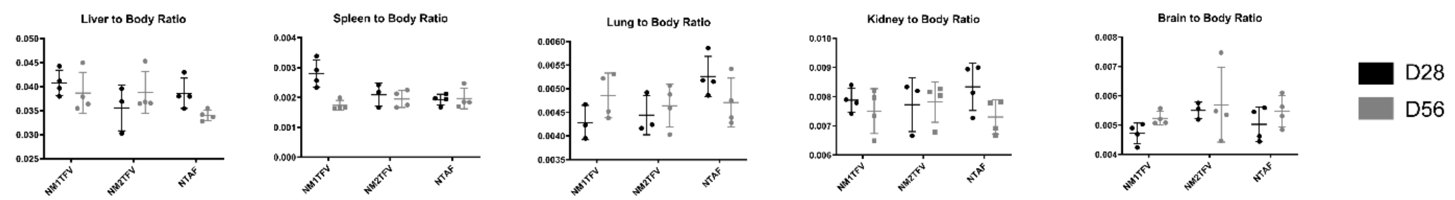


b

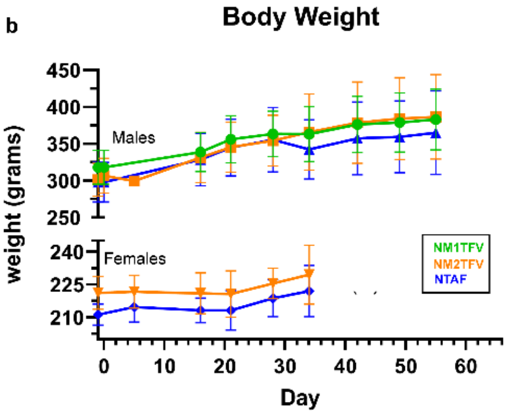


c

| | NM1TFV | NM2TFV | NTAF | Untreated | Range | |
|---|---|---|---|---|---|---|
| **WBC** | 8.68 ± 3.93 | 11.37 ± 3.76 | 8.56 ± 2.58 | 7.03 ± 2.97 | 2.1- 19.5 | $10^9$/ l |
| LYM | 6.37 ± 2.81 | 8.60 ± 2.92 | 6.97 ± 1.90 | 4.93 ± 2.6 | 2- 14.1 | $10^9$/ l |
| MON | 0.44 ± 0.58 | 0.15 ± 0.22 | 0.51 ± 0.41 | 0.19 ± 0.12 | 0- 0.98 | $10^9$/ l |
| NEU | 1.88 ± 1.32 | 2.62 ± 0.65 | 1.08 ± 0.42 | 2.09 ± 1.18 | 0.1- 5.4 | $10^9$/ l |
| EOS | | | | ± | | $10^9$/ l |
| BAS | | | | ± | | $10^9$/ l |
| LY% | 74.13 ± 5.24 | 76.03 ± 40.33 | 81.90 ± 4.33 | 67.2 ± 0.43 | 55- 97 | % |
| MO% | 4.48 ± 4.21 | 1.58 ± 1.60 | 5.23 ± 3.42 | 3.37 ± 3.05 | 0- 5 | % |
| NE% | 21.38 ± 8.58 | 22.35 ± 7.68 | 12.83 ± 3.24 | 29.4 ± 10.6 | 2- 31.0 | % |
| EO% | | | | | | % |
| BA% | | | | | | % |
| **RBC** | 10.95 ± 1.64 | 9.98 ± 5.12 | 10.15 ± 1.56 | 8.97 ± 0.42 | 5.3- 10 | $10^{12}$/ l |
| HGB | 16.25 ± 2.41 | 14.83 ± 7.61 | 15.97 ± 1.22 | 15.9 ± 0.95 | 14- 18 | g/dl |
| HCT | 52.68 ± 8.75 | 48.71 ± 24.29 | 48.76 ± 8.71 | 45.23 ± 1.9 | 35- 52 | % |
| MCV | 48.00 ± 2.16 | 48.75 ± 26.68 | 47.67 ± 2.08 | 50.33 ± 1.53 | 50- 62 | fl |
| MCH | 14.85 ± 0.30 | 14.83 ± 8.39 | 15.87 ± 1.61 | 17.73 ± 0.55 | 16- 23 | pg |
| MCHC | 30.85 ± 0.86 | 30.53 ± 17.22 | 33.17 ± 3.74 | 35.17 ± 0.57 | 31- 40 | g/dl |
| RDWc | 19.28 ± 0.38 | 18.33 ± 10.65 | 17.97 ± 0.85 | 18.8 ± 0.35 | | % |
| **PLT** | 631.00 ± 152.87 | 571.25 ± 260.52 | 521.33 ± 294.53 | 344 ± 14.73 | 500- 1370 | $10^9$/ l |
| PCT | 0.45 ± 0.11 | 0.42 ± 0.19 | 0.37 ± 0.23 | 0.24 ± 0.03 | | % |
| MPV | 7.05 ± 0.40 | 5.83 ± 3.54 | 7.03 ± 0.45 | 6.97 ± 0.76 | | fl |
| PDWc | 35.48 ± 3.44 | 35.38 ± 18.47 | 33.00 ± 0.80 | 34.53 ± 4.29 | | % |

**Supplementary Figure 13. Toxicology.** Toxicity was assessed in SD rats after NM1TFV, NM2TFV, and NTAF treatment. **(a)** Organ to body weight ratios were determined on the days of sacrifice (days 28 in black, and 56 in grey), and **(b)** animal weights were recorded weekly (green= NM1TFV, orange= NM2TFV, blue= NTAF). Values reported are the mean ± SEM of 4 animals. **(c)** Plasma was collected for CBCs, controls were age-matched untreated mice. Values reported are the SEM of 4 replicates.

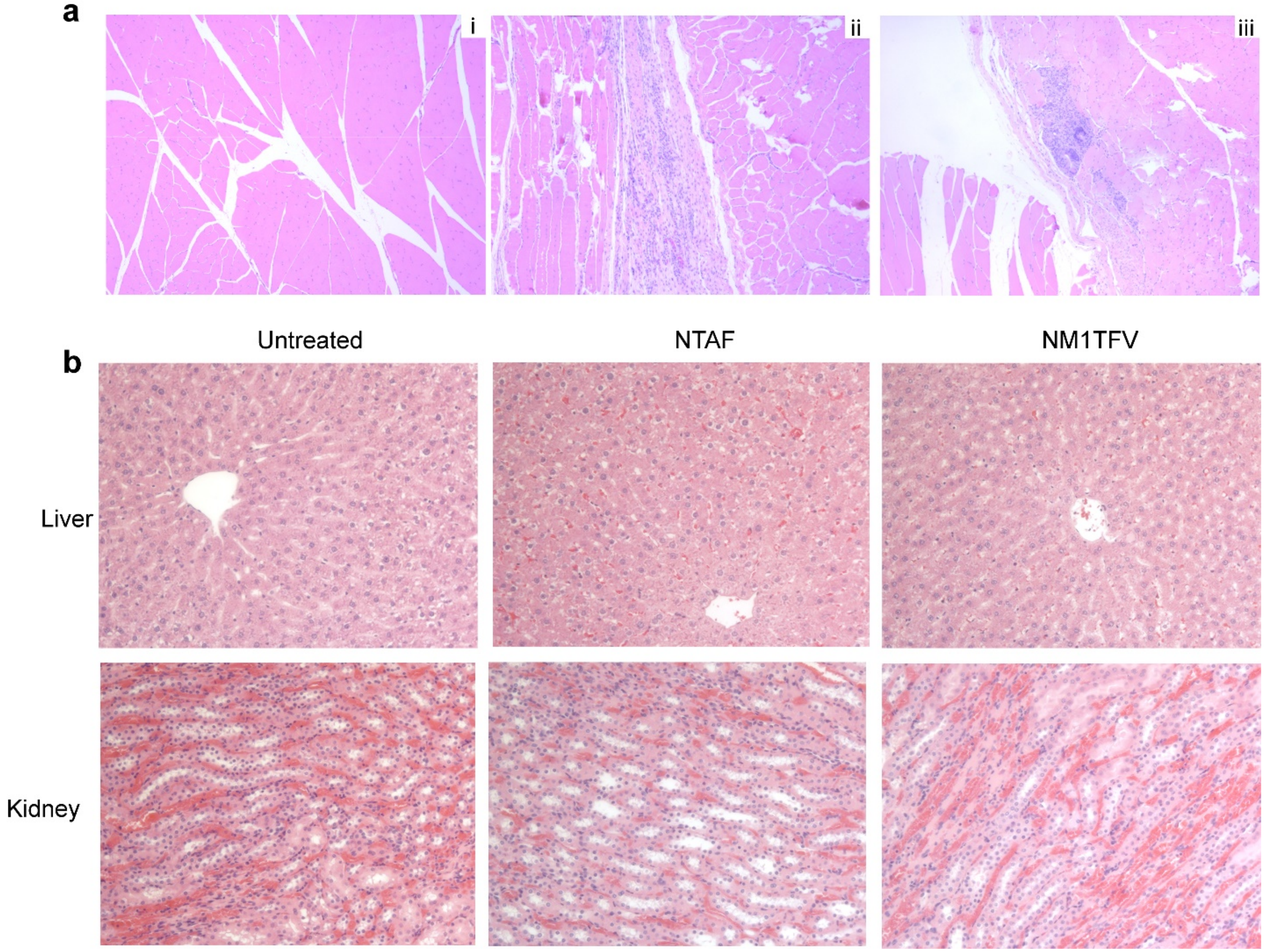


**Supplementary Figure 14. Tissue Histopathology.** Following a single IM dose of NM1TFV or NTAF male SD rats were sacrificed on days 28 and 56. 5-μm sections of paraffin-embedded **(a)** muscle, **(b)** liver and kidney were stained with hematoxylin, and images were captured with a ×20 objective lens. **(a)** H&E staining of the site of injection in NTAF treated animals at days (**ii**) 28 and (**iii**) 56 post treatment were performed and compared to control untreated muscle (**i**). **(b)** 5 μm sections of paraffin-embedded liver and kidney were stained with hematoxylin, which revealed no abnormal pathology. Study was performed once; images are representative of the histological findings from 3 biological replicates.

**pH 2**

| Time (hr) | Discovery? | P value | Mean of NTAF | Mean of NM1TFV | Difference | SE of difference | t ratio | df | q value |
|---|---|---|---|---|---|---|---|---|---|
| 0 | | | 100 | 100 | 0 | 0 | | | |
| 0.0833 | | | 100 | 100 | 0 | 0 | | | |
| 0.5 | No | 0.459001 | 94.36 | 100.3 | -5.94 | 7.257 | 0.8186 | 4 | 0.309061 |
| 1 | No | 0.339067 | 89.91 | 98.86 | -8.95 | 8.251 | 1.085 | 4 | 0.298496 |
| 2 | No | 0.067273 | 87.64 | 95.41 | -7.77 | 3.117 | 2.493 | 4 | 0.090594 |
| 6 | Yes | 0.000104 | 66.48 | 92.47 | -25.99 | 1.69 | 15.38 | 4 | 0.000315 |
| 12 | Yes | 0.000156 | 59.44 | 97.65 | -38.21 | 2.752 | 13.88 | 4 | 0.000315 |
| 24 | No | 0.369426 | 52.26 | 48.79 | 3.47 | 3.434 | 1.01 | 4 | 0.298496 |

**pH 6**

| Time (hr) | Discovery? | P value | Mean of NTAF | Mean of NM1TFV | Difference | SE of difference | t ratio | df | q value |
|---|---|---|---|---|---|---|---|---|---|
| 0 | | | 100 | 100 | 0 | 0 | | | |
| 0.0833 | | | 100 | 100 | 0 | 0 | | | |
| 0.5 | No | 0.077342 | 86.14 | 98.88 | -12.74 | 5.39 | 2.364 | 4 | 0.095391 |
| 1 | No | 0.68173 | 80.57 | 82.24 | -1.67 | 3.783 | 0.4414 | 4 | 0.573789 |
| 2 | No | 0.080671 | 77.85 | 74.26 | 3.59 | 1.544 | 2.325 | 4 | 0.095391 |
| 6 | No | 0.094447 | 58.27 | 76.92 | -18.65 | 8.544 | 2.183 | 4 | 0.095391 |
| 12 | No | 0.012645 | 47.15 | 78.56 | -31.41 | 7.305 | 4.3 | 4 | 0.031929 |
| 24 | Yes | 0.000191 | 15.19 | 47.31 | -32.12 | 2.435 | 13.19 | 4 | 0.000964 |

**pH 7**

| Time (hr) | Discovery? | P value | Mean of NTAF | Mean of NM1TFV | Difference | SE of difference | t ratio | df | q value |
|---|---|---|---|---|---|---|---|---|---|
| 0 | | | 100 | 100 | 0 | 0 | | | |
| 0.0833 | | | 100 | 100 | 0 | 0 | | | |
| 0.5 | No | 0.966899 | 86.73 | 86.93 | -0.2 | 4.53 | 0.04415 | 4 | 0.976568 |
| 1 | No | 0.105015 | 79.08 | 87.23 | -8.15 | 3.902 | 2.088 | 4 | 0.318195 |
| 2 | No | 0.017605 | 72.42 | 85.37 | -12.95 | 3.324 | 3.896 | 4 | 0.106686 |
| 6 | No | 0.477185 | 48.33 | 55.18 | -6.85 | 8.744 | 0.7834 | 4 | 0.722935 |
| 12 | No | 0.682619 | 43.96 | 47.43 | -3.47 | 7.885 | 0.4401 | 4 | 0.827335 |
| 24 | No | 0.174356 | 20.4 | 38.23 | -17.83 | 10.81 | 1.65 | 4 | 0.352199 |

**pH 8**

| Time (hr) | Discovery? | P value | Mean of NTAF | Mean of NM1TFV | Difference | SE of difference | t ratio | df | q value |
|---|---|---|---|---|---|---|---|---|---|
| 0 | | | 100 | 100 | 0 | 0 | | | |
| 0.0833 | | | 100 | 100 | 0 | 0 | | | |
| 0.5 | Yes | 0.001787 | 81.49 | 101.6 | -20.12 | 2.722 | 7.391 | 4 | 0.002444 |
| 1 | Yes | 0.002558 | 76.32 | 101.4 | -25.08 | 3.734 | 6.717 | 4 | 0.002584 |
| 2 | Yes | 0.002017 | 60.86 | 94.5 | -33.64 | 4.7 | 7.157 | 4 | 0.002444 |
| 6 | Yes | 0.000048 | 25.76 | 63.3 | -37.54 | 2.002 | 18.75 | 4 | 0.000096 |
| 12 | Yes | <0.000001 | 10.38 | 35.28 | -24.9 | 0.3111 | 80.03 | 4 | <0.000001 |
| 24 | Yes | 0.000002 | 1.48 | 38.9 | -37.42 | 0.8415 | 44.47 | 4 | 0.000005 |

**pH 10.3**

| Time (hr) | Discovery? | P value | Mean of NTAF | Mean of NM1TFV | Difference | SE of difference | t ratio | df | q value |
|---|---|---|---|---|---|---|---|---|---|
| 0 | | | 100 | 100 | 0 | 0 | | | |
| 0.0833 | | | 100 | 100 | 0 | 0 | | | |
| 0.5 | Yes | 0.006692 | 63.59 | 98.3 | -34.71 | 6.725 | 5.161 | 4 | 0.001352 |
| 1 | Yes | 0.000011 | 25.18 | 90.93 | -65.75 | 2.433 | 27.03 | 4 | 0.000011 |
| 2 | Yes | 0.000034 | 8.64 | 66.5 | -57.86 | 2.844 | 20.34 | 4 | 0.000013 |
| 6 | Yes | 0.000039 | 0 | 23.66 | -23.66 | 1.2 | 19.72 | 4 | 0.000013 |
| 12 | Yes | 0.002167 | 0 | 19.31 | -19.31 | 2.75 | 7.022 | 4 | 0.000547 |
| 24 | Yes | 0.015874 | 0 | 10.41 | -10.41 | 2.59 | 4.019 | 4 | 0.002672 |

**Supplementary Table 1. Statistical Analysis of pH Stability Study.** An unpaired t-test assuming individual variance for each row, followed by multiple comparisons made using the two-stage setup procedure of Benjamin, Krieger and Yekutieli with a false discovery rate (FDR) of 1.00% was used to compare the mean prodrug concentration of NM1TFV compared to NTAF in various pHs from 0-24 hours. “Yes” in the Discovery column indicates timepoints where the means of NM1TFV and NTAF reach statistical significance.

## Supplementary Methods

### Nuclear Magnetic Resonance (NMR) of TFV ProTides

Successful synthesis of the prodrugs was confirmed by $^{1}H$ and $^{31}P$ NMR spectroscopy using a Bruker Avance-III HD operating at 500 MHz and a magnetic field strength of 11.7 T. **M1TFV**: $^{1}H$ NMR (500 MHz, $CDCl_3$): 8.32 (2s, 1H), 7.92 (2s, 1H), 7.04-7.38 (m, 7H), 6.93-7.02 (m, 2H), 5.68 (d, $J$ = 12.9 Hz, 1H), 4.25-4.13 (m, 2H), 3.80-4.15 (m, 4H), 3.34-3.65 (m, 2H), 2.86-2.94 (m, 2H), 1.82 (b, 3H), 1.51 (b, 2H), 1.21-1.35 (m, 37H), 1.15 (d, $J$ = 6.2 Hz, 3H), 0.87 (t, $J$ = 6.9 Hz, 3H). $^{13}C$ NMR (125 MHz, $CDCl_3$): δ 172.6, 155.6, 152.9, 150.2, 150.1, 149.9, 141.7, 135.9, 129.6, 129.5, 129.4, 128.4, 126.9, 124.9, 120.6, 120.5, 120.4, 119.2, 76.6, 76.1, 65.6, 64.9, 63.8, 63.7, 55.0, 54.9, 54.7, 50.4, 48.3, 48.2, 48.1, 41.2, 41.0, 40.5, 31.9, 30.9, 29.7, 29.6, 29.5, 29.3, 29.2, 28.4, 25.8, 22.6, 16.4, 14.1; $^{31}P$ NMR (202 MHz, $CDCl_3$); 20.9, 22.1. MALDI-TOF m/z: $[M + H]^+$: calcd. for $C_{46}H_{72}N_6O_5P^+$, 819.53 (100%), 820.53 (49.8%), 821.54 (12.1%); found, 819.42. **M2TFV**: $^{1}H$ NMR (500 MHz, $CDCl_3$): 8.34 (2s, 1H), 7.98 (2s, 1H), 7.31 (t, $J$ = 7.9 Hz, 1H), 7.21 (t, $J$ = 7.8 Hz, 1H), 7.14 (d, $J$ = 7.8 Hz, 1H), 7.10 (t, $J$ = 7.3 Hz, 1H), 6.99 (d, $J$ = 7.3 Hz, 1H), 5.66 (d, $J$ = 8.9 Hz, 1H), 4.34 (dd, $J$ = 14.4, 2.9 Hz, 1H), 3.86-4.20 (m, 5H), 3.55-3.73 (m, 1H), 3.49 (t, $J$ = 10.4 Hz, 1H), 1.53-1.68 (m, 2H), 1.20-1.38 (m, 42H), 0.87 (t, $J$ = 6.9 Hz, 3H). $^{13}C$ NMR (125 MHz, $CDCl_3$): δ 173.8, 155.6, 152.9, 150.5, 150.1, 150.0, 141.7, 129.7, 129.6, 129.5, 129.2, 124.9, 120.8, 120.4, 119.2, 76.6, 76.2, 65.6, 65.3, 64.9, 63.8, 49.9, 49.6, 48.3, 40.9, 31.9, 30.9, 29.7, 29.6, 29.5, 29.4, 29.2, 28.6, 28.5, 25.8, 22.6, 21.6, 20.9, 16.4, 14.1; $^{31}P$ NMR (202 MHz, $CDCl_3$); 20.7, 22.2. MALDI-TOF m/z: $[M + H]^+$: calcd. for $C_{40}H_{68}N_6O_5P^+$, 743.50 (100%), 744.50 (43.3%), 745.51 (9.1%); found, 743.36.
