# Supplementary material for: Transformation of tenofovir into stable ProTide nanocrystals with long-acting pharmacokinetic profiles
Source: Nat Commun. 2021 Sep 16;12:5458. doi: 10.1038/s41467-021-25690-5 (PMC8445934; doi:10.1038/s41467-021-25690-5)
Supplement: Supplementary file 3 — Reporting Summary [file 41467_2021_25690_MOESM3_ESM.pdf]

nature research

Corresponding author(s): Benson J. Edagwa
Last updated by author(s): Aug 16, 2021

Reporting Summary

Nature Research wishes to improve the reproducibility of the work that we publish. This form provides structure for consistency and transparency in reporting. For further information on Nature Research policies, see our Editorial Policies and the Editorial Policy Checklist.

Statistics

For all statistical analyses, confirm that the following items are present in the figure legend, table legend, main text, or Methods section.

n/a	Confirmed	
☐	☒	The exact sample size (*n*) for each experimental group/condition, given as a discrete number and unit of measurement
☐	☒	A statement on whether measurements were taken from distinct samples or whether the same sample was measured repeatedly
☐	☒	The statistical test(s) used AND whether they are one- or two-sided<br>*Only common tests should be described solely by name; describe more complex techniques in the Methods section.*
☒	☐	A description of all covariates tested
☐	☒	A description of any assumptions or corrections, such as tests of normality and adjustment for multiple comparisons
☐	☒	A full description of the statistical parameters including central tendency (e.g. means) or other basic estimates (e.g. regression coefficient) AND variation (e.g. standard deviation) or associated estimates of uncertainty (e.g. confidence intervals)
☐	☒	For null hypothesis testing, the test statistic (e.g. *F*, *t*, *r*) with confidence intervals, effect sizes, degrees of freedom and *P* value noted<br>*Give P values as exact values whenever suitable.*
☒	☐	For Bayesian analysis, information on the choice of priors and Markov chain Monte Carlo settings
☒	☐	For hierarchical and complex designs, identification of the appropriate level for tests and full reporting of outcomes
☐	☒	Estimates of effect sizes (e.g. Cohen's *d*, Pearson's *r*), indicating how they were calculated

Our web collection on statistics for biologists contains articles on many of the points above.

Software and code

Policy information about availability of computer code

Data collection	MassLynx V4.1 software (Waters, Milford, MA)
Data analysis	MassLynx V4.1 software (Waters, Milford, MA); Microsoft Excel V16.45; GraphPad Prism V9.0.0.0

For manuscripts utilizing custom algorithms or software that are central to the research but not yet described in published literature, software must be made available to editors and reviewers. We strongly encourage code deposition in a community repository (e.g. GitHub). See the Nature Research guidelines for submitting code & software for further information.

Data

Policy information about availability of data

All manuscripts must include a data availability statement. This statement should provide the following information, where applicable:
- Accession codes, unique identifiers, or web links for publicly available datasets
- A list of figures that have associated raw data
- A description of any restrictions on data availability

Datasets corresponding to each figure are accessible via: 10.6084/m9.figshare.15168909

Field-specific reporting

Life sciences study design

All studies must disclose on these points even when the disclosure is negative.

Sample size	For in vitro uptake, retention, antiviral efficacy studies no statistical method was used to predetermine sample size. However, for in vivo studies sample sizes chosen were sufficient to determine significance, with reproducible statistically significant differences between experimental conditions. For comparing two groups for PK analysis, four animals/group will provide 90% power at the 0.05 level of significance to detect a difference of 2.0 standard deviations using a t-test. For animal studies, sample sizes were determined in order to provide statistical power while also meeting cost and ethical criteria for animal use.
Data exclusions	Exclusion criteria was predetermined. Using the ROUT method, outliers beyond the 99% confidence interval of the mean were excluded. No data were determined to fit this criteria, therefore no data points were excluded from the current reported values.
Replication	All attempts to reproduce the experimental findings were successful. For chemical synthesis, characterization, and formulation production, experiments were repeated independently a minimum of three times with similar results. For in vitro cellular assays, experiments were repeated independently a minimum two times with similar results. For prodrug hydrolysis studies, experiments were conducted once with N=3. Rat studies were conducted once with an N = 4 animals per group.
Randomization	For all studies, samples/cells/animals were randomly allocated into experimental groups at the beginning of each study.
Blinding	In vitro studies were not blinded (conducting in vitro experiments or sample collection) because only one investigator was involved in conducting those studies. However, studies were designed such that histological and PK data collection and analysis were blinded. Personnel involved in tissue histology sample preparation, mass-spectrometry, data collection, and sample analysis were blinded as to treatments.

Reporting for specific materials, systems and methods

We require information from authors about some types of materials, experimental systems and methods used in many studies. Here, indicate whether each material, system or method listed is relevant to your study. If you are not sure if a list item applies to your research, read the appropriate section before selecting a response.

Materials & experimental systems

n/a	Involved in the study
☐	☒ Antibodies
☐	☒ Eukaryotic cell lines
☒	☐ Palaeontology and archaeology
☐	☒ Animals and other organisms
☐	☒ Human research participants
☒	☐ Clinical data
☒	☐ Dual use research of concern

Methods

n/a	Involved in the study
☒	☐ ChIP-seq
☒	☐ Flow cytometry
☒	☐ MRI-based neuroimaging

Antibodies

Antibodies used	The polymer-based HRP-conjugated goat anti-mouse EnVision+ secondary (cat. K4000; lot 10122735) was purchased from Dako (Carpinteria, CA), now Agilent Technologies (Santa Clara, CA). The monoclonal mouse anti-human HIV-1p24 [cat. M0857; clone Kal-1; Dako, Carpenteria, CA, USA), (Kaluza, G. et al. (1992)].
Validation	HIV-1p24 antibody provides excellent specificity, high lot to lot consistency, and certified manufacturing facilities guarantee full quality control (per company website). HLA-DP/DQ/DR antibody provides excellent specificity, high lot to lot consistency, optimized for immunohistochemistry (IHC) with validated protocols, and certified manufacturing facilities guarantee full quality control (per company website).

Eukaryotic cell lines

Policy information about cell lines

Cell line source(s)	CEM-SS Cells (ARP-776 , NIH Reagent Program)
Authentication	For CEM-ss cells, lot specific certificate of analysis was used to confirm the cell line. No in house authentication was performed.
Mycoplasma contamination	Cells obtained from elutriation were negative for mycoplasma. For CEM-ss cells, the agency assessed the cell line for Mycoplasma before shipment. Following expansion of the cell line in our laboratory, the CEM-ss cells were batch-tested for mycoplasma contamination before cryo-preservation (tested negative). No mycoplasma testing was performed after defrosting.

Commonly misidentified lines (See ICLAC register)	No commonly misidentified cell lines were used.

Animals and other organisms

Policy information about studies involving animals; ARRIVE guidelines recommended for reporting animal research

Laboratory animals	Rats: Sprague Dawley, male and female, 12 weeks
Wild animals	The study did not involve wild animals.
Field-collected samples	The study did not involve samples collected from the field.
Ethics oversight	All experimental protocols involving the use of laboratory animals were approved by the UNMC Animal Care and Use Committees (IACUC) ensuring the ethical care and use of laboratory animals in experimental research. All animal studies were performed in compliance with institutional policies and NIH guidelines for laboratory animal housing and care in American Animal Association and Laboratory Animal Care (AAALAC) accredited facilities.

Note that full information on the approval of the study protocol must also be provided in the manuscript.

Human research participants

Policy information about studies involving human research participants

Population characteristics	HIV-1/2 and hepatitis B seronegative
Recruitment	The investigators of this study were not involved in the recruitment of research participants.
Ethics oversight	Human monocytes were isolated by leukapheresis from HIV-1/2 and hepatitis B seronegative donors according to a UNMC Institutional Review Board (IRB) exempt protocol. All donors gave informed consent for the use of the deidentified material. Cells obtained from elutriation were negative for mycoplasma contamination.

Note that full information on the approval of the study protocol must also be provided in the manuscript.
